# Mechanical Regulation of Protein Translation in the Cardiovascular System

**DOI:** 10.3389/fcell.2020.00034

**Published:** 2020-01-31

**Authors:** Lisa J. Simpson, John S. Reader, Ellie Tzima

**Affiliations:** Division of Cardiovascular Medicine, Radcliffe Department of Medicine, Wellcome Centre for Human Genetics, University of Oxford, Oxford, United Kingdom

**Keywords:** mechanotransduction, protein translation, cardiac, endothelial, shear stress, pressure overload

## Abstract

The cardiovascular system can sense and adapt to changes in mechanical stimuli by remodeling the physical properties of the heart and blood vessels in order to maintain homeostasis. Imbalances in mechanical forces and/or impaired sensing are now not only implicated but are, in some cases, considered to be drivers for the development and progression of cardiovascular disease. There is now growing evidence to highlight the role of mechanical forces in the regulation of protein translation pathways. The canonical mechanism of protein synthesis typically involves transcription and translation. Protein translation occurs globally throughout the cell to maintain general function but localized protein synthesis allows for precise spatiotemporal control of protein translation. This Review will cover studies on the role of biomechanical stress -induced translational control in the heart (often in the context of physiological and pathological hypertrophy). We will also discuss the much less studied effects of mechanical forces in regulating protein translation in the vasculature. Understanding how the mechanical environment influences protein translational mechanisms in the cardiovascular system, will help to inform disease pathogenesis and potential areas of therapeutic intervention.

## Forces in Biology

Mechanical forces can occur on the whole body to microscopic scale. During development, mechanical forces govern cell shape and migration and hence orchestrate the growth of multicellular biological tissues (Mammoto and Ingber, 2010; LeGoff and Lecuit, 2016). Mechanical signals drive organogenesis in the late stages of embryonic development in nearly every system. Force drives the formation of the vasculature (Lucitti et al., 2007), lungs (Gutierrez et al., 2003), brain (Anava et al., 2009), musculoskeletal system (Stokes et al., 2002; Kahn et al., 2009) hematopoietic system (Adamo et al., 2009; North et al., 2009) and the heart (Hove et al., 2003; Forouhar et al., 2006). Impaired force sensing or altered mechanotransduction signaling is linked to defects in development of tissues and organs in addition to disease in later life such as cardiovascular diseases and cancer (Jaalouk and Lammerding, 2009).

External forces such as those imposed by gravity and exercise influence musculoskeletal growth and strength. Skeletal muscle requires mechanical load which acts to upregulate protein synthesis and promote myocyte growth and maintenance. Bone growth and metabolism requires frequent pressure and tensile forces generated through skeletal muscle contractions and gravity (Kohrt et al., 2009). Mechanical loading is essential for musculoskeletal homeostasis as withdrawal of force leads to regression and atrophy of these tissues (reviewed well by Felsenthal and Zelzer, 2017). Atrophy is often observed with aging but also in environments such as microgravity in space where force bearing on the skeleton is lower than on Earth (LeBlanc et al., 2007; Amin, 2010). These changes involve alterations in protein synthesis mechanisms, which, in the absence of mechanical stimuli, contribute to reduced growth and cell turnover.

Respiration is another highly mechanical process requiring repeated expansion and subsequent deflation of the lungs in order to oxygenate blood and remove gaseous waste from the body. This vital continual loop generates an array of mechanical forces within the pulmonary system, such as longitudinal or circumferential stretch, surface tension on the alveoli cells of the lungs or fluid shear stress within the pulmonary vasculature caused by blood flow (Breen et al., 1999). Increased ventilation is associated with remodeling of the lungs that involves increases in both protein and DNA as a result of mechanical force (Gutierrez et al., 2003). Mechanical forces are also implicated in lung diseases, for example, force imbalances arising from pulmonary hypertension can remodel the pulmonary vasculature and induce smooth muscle and fibroblast proliferation in conjunction with increased collagen and elastin protein synthesis and gene expression (Wirtz and Dobbs, 2000). Disturbed mechanical forces exerted on pulmonary vascular endothelial cells induces changes in protein synthesis and expression of pro-inflammatory molecules such as IL-8, TNF-α and CXCL5 (Tang et al., 2016).

## Mechanics in the Cardiovascular System

Atherosclerosis is characterized as a chronic inflammatory disorder resulting from the accumulation of fatty deposits in the arterial intima which can form plaques occluding blood flow. This disease is highly focal in nature as a result of differential mechanical forces exerted by blood flow in different regions of the vascular network. Briefly, disturbed and complex blood flow profiles arise where blood vessels branch or curve sharply and this primes these areas to have low but chronic inflammation, leaving them more vulnerable to plaque accumulation (Davies, 1995; Hahn and Schwartz, 2009). Conversely, straight regions of the vasculature experience smooth, uniform blood flow which promotes anti-inflammatory pathways and thus keeps them relatively protected from atherosclerotic plaques. Atherosclerotic lesions leave arteries thicker and less compliant therefore they cannot modulate or adapt to changes in blood flow. Rapid changes in shear stress gradients at lesion sites has been shown to be the primary driver of platelet aggregation and activation which increases plaque accumulation (Nesbitt et al., 2009). Disturbed blood flow at injury sites can increase risk of plaque rupture and thrombotic complications downstream. This can be fatal if it occurs in the coronary vessels of the heart. In addition to atherosclerosis, changes to blood flow parameters in the aorta can promote abnormal swelling and weakening of the vessel wall known as aortic aneurysms. Under mechanical strain, these bulges will eventually rupture, leading to potentially fatal complications (Bäck et al., 2013).

The organ that is perhaps the most influenced by biomechanical forces is the heart. The heart beats continuously to transport oxygenated blood and nutrients to the rest of the body to maintain normal organ function. The activity and integrity of the heart itself is highly influenced by biomechanics and mechanical stress is a critical mediator of cardiomyocyte function and extracellular matrix composition (Voorhees and Han, 2015). Biomechanical forces regulate the activity and function of the cells of the heart: cardiomyocytes, fibroblasts, and the vascular cells of the coronary blood vessels (Hahn and Schwartz, 2009; Voorhees and Han, 2015; van Putten et al., 2016; Herum et al., 2017). In response to biomechanical stress, cardiomyocytes undergo hypertrophic growth (Hannan et al., 2003). Hypertrophic adaptive remodeling can occur under physiological settings such as exercise or during pregnancy where the heart undergoes compensatory hypertrophy to deal with increased mechanical load in order to maintain cardiac function and output (Heineke and Molkentin, 2006). In response to chronic endurance exercise undertaken by elite athletes, the heart must remodel to handle the considerable increase in mechanical load (George et al., 2012). Physiological hypertrophy seen in the athlete’s heart is typically not associated with myocyte damage, although some studies have shown myocardial death during intense exercise as well as fibrosis in long-term endurance athletes (La Gerche et al., 2012; Galderisi et al., 2015). Nevertheless, most elite athletes will present a healthy physiological adaptation to prolonged bouts of intense exercise that can be distinguished from pathological hypertrophy to pressure overload. Under pathological settings, such as hypertension, the heart deals with sustained, chronic levels of mechanical strain and this can lead to persistent activation of protein synthesis pathways such as mammalian target of rapamycin (mTOR) which regulate cardiomyocyte growth (Heineke and Molkentin, 2006). This hypertrophic remodeling response is chronic and can ultimately result in heart failure (Lyon et al., 2015).

## Protein Translation and Cardiovascular Function

Protein translation is a highly conserved and tightly regulated process which is fundamental for cellular homeostasis. The canonical mechanism of protein synthesis typically involves two major steps: transcription of a messenger RNA (mRNA) transcript in the nucleus and translation of this mRNA into a protein by the translational machinery in the cytoplasm (Clancy and Brown, 2008). Protein translation occurs globally throughout the cell to maintain general function but localized or polarized protein synthesis occurring for example at the leading edge of migrating cells (Katz et al., 2012), allows for efficient translation of specific proteins required for cell motility in the correct location. It is important to emphasize that mRNA levels do not always correlate to protein expression levels and this disconnect is a result of post transcriptional mechanisms (Spriggs et al., 2010). Having this additional level of translational control enables cells to rapidly respond and adapt to changing micro-environmental conditions.

Translation is segmented into four stages: initiation, elongation, termination and ribosome recycling. Modulation of translation typically occurs at the initiation stage which requires the co-ordination of many translational factors and ribosomal subunits (Sonenberg and Hinnebusch, 2009). Eukaryotic initiation factors (eIFs) are involved in mediating the start of translation through assembly of initiation complex on the 40S ribosomal subunit and chaperoning of the 60S subunit to join the 40S (Sonenberg and Hinnebusch, 2009). The activity of eIFs are controlled via phosphorylation and the most common mechanism for switching off global translation is through phosphorylation of eIF2α subunit at its Serine 51 (Jackson et al., 2010). This highlights the importance of translation modulation under conditions of cell stress or when the cell needs to conserve energy. Having the ability to switch off global translation and shift the proteomic landscape to synthesize specific proteins required to maintain cellular function is critical for cell survival.

**FIGURE 1:**
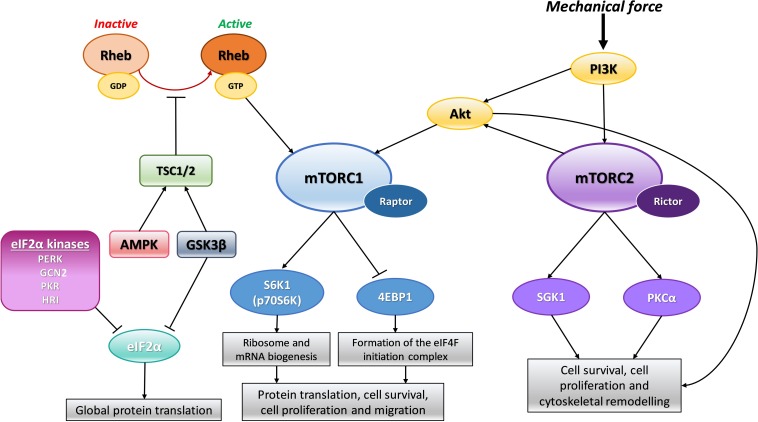
Schematic representation of mTOR signaling. In response to mechanical force, PI3K activates Akt and mTORC2 directly. mTORC2 can further activate Akt which can also directly activate mTORC1. Rheb can directly activate mTORC1 when in its active GTP bound form. Activation of mTORC1 positively regulates S6K1/p70S6K leading to downstream ribosome and mRNA biogenesis. In addition, activation of mTORC1 also negatively regulates 4EBP1 allowing for the formation of the eIF4F translation initiation complex. This combined signaling promotes protein synthesis and cell growth. When mTORC2 is activated it also promotes cell growth and survival through its downstream effectors SGK1 and PKCα. Adaptor proteins, Raptor and Rictor, are specific to mTORC1 and mTORC2, respectively, and are required for active signal transduction. The TSC1/2 complex can prevent mTORC1 activation by Rheb by keeping Rheb in its inactive GDP form. The TSC1/2 complex can be activated by both AMPK and GSK3β signaling to dampen mTORC1 activity in times of cellular stress. GSK3β can also inhibit eIF2α mediated protein translation again to reduce global protein synthesis during cellular stress. In addition, there are four kinases which can phosphorylate and inactivate eIF2α mediated protein translation under distinct stress conditions: PERK, GCN2, PKR and HRI. Phosphoinositide-3-kinase–protein kinase B/Akt (PI3K-PKB/Akt), Mammalian target of rapamycin (mTOR), Tuberous sclerosis protein (TSC), 5′ adenosine monophosphate-activated protein kinase (AMPK), Glycogen synthase kinase 3β (GSK3β), Ras homolog enriched in brain (Rheb), Eukaryotic initiation factor 2α (eIF2α), Protein kinase R (PKR)-like endoplasmic reticulum kinase (PERK), General control non-derepressible 2 (GCN2), Protein kinase RNA-activated (PKR), Heme-regulated inhibitor kinase (HRI), Ribosomal protein S6 kinase beta-1 (S6K1) or p70S6 kinase (p70S6K), Eukaryotic translation initiation factor 4E (eIF4E)-binding protein 1 (4EBP1), Serine/threonine-protein kinase Sgk1 also known as serum and glucocorticoid-regulated kinase 1 (SGK1), Protein kinase C alpha (PKCα).

The highly conserved regulatory pathway, mTOR plays a crucial role in many processes including transcription and protein translation, ribosomal and mitochondrial biogenesis, and cell growth and division (Sciarretta et al., 2014). mTOR is a serine/threonine protein kinase, part of the phosphoinositide 3-kinase (PI3K) family, which interacts with many adaptor proteins to form two distinct signaling complexes, namely mTORC1 and mTORC2. These complexes were distinguished by their relative sensitivity to Rapamycin, which inhibits mTORC1 signaling without disrupting mTORC2 signaling. Broadly, mTORC1 regulates protein synthesis, cell growth and proliferation, cell metabolism and stress responses, whereas mTORC2 regulates cell survival, cytoskeletal organization and polarity (Figure 1). Both complexes are relatively large with multiple adaptor proteins which give them their unique signaling identity. Of the two complexes, mTORC1 has been more extensively studied and its upstream inputs and downstream targets are better understood than that of mTORC2, in the cardiovascular system and the rest of the body (Sciarretta et al., 2018).

The major downstream substrates of mTORC1 related to protein synthesis are S6 kinase 1 (S6K1) and eukaryotic ignition factor 4E (eIF4E)-binding protein (4E-BP1) and these have been thoroughly studied (Shin et al., 2011). Once activated, mTORC1 phosphorylates and in turn activates S6K1 which stimulates mRNA biogenesis and the protein translation machinery. mTORC1 negatively regulates 4E-BP1 and in doing so this allows for the formation of the eIF4F initiation complex that promotes the canonical cap-dependent pathway of protein translation. Protein kinase B, or Akt, can directly activate mTOR through phosphorylation whilst also repressing the endogenous mTORC1 inhibitor, PRAS40, and thereby augment mTOR downstream effects. The 5′ adenosine monophosphate-activated protein kinase (AMPK) pathway is a well-established negative regulator of mTORC1 activity. AMPK is normally activated in times of cellular stress e.g., when nutrients, amino acids and energy are scarce. Under stressed conditions, AMPK will stimulate the tuberous sclerosis protein (TSC) 1/TSC2 complex which can inhibit mTORC1 signaling indirectly by converting mTORC1 activator Ras homolog enriched in brain (Rheb) into its inactive GDP-bound form. The active GTP-bound state of Rheb normally directly interacts with and promotes mTORC1’s kinase functions (Laplante and Sabatini, 2012). In addition to AMPK, glycogen synthase kinase (GSK) 3β is a potent activator of the TSC1/TSC2 complex and so can also contribute to dampening of mTORC1 activity during times of cellular stress (see Figure 1 for summary of signaling). It is well established that mTOR signaling can be activated by amino acids, stress, oxygen, energy status and growth factors such as insulin (Laplante and Sabatini, 2012). There is also evidence indicating mechanical force can stimulate mTOR signaling (Kraiss et al., 2000; Guo et al., 2007; Hornberger, 2011; Philip et al., 2011; Jacobs et al., 2017; Vion et al., 2017) and this will be discussed in more detail below.

## Protein Translation in Response to Biomechanical Forces in the Heart

Force-derived signaling regulates the development of cardiomyopathy and left ventricular remodeling following an infarct by contributing to tissue fibrosis and scarring. Elevated stress and pressure overload on the heart in conditions such as hypertension and valvular disease can promote ventricular hypertrophy and diastolic heart failure (Merino et al., 2018). The most common model used to mimic human cardiovascular disease and elucidate mechanisms of cardiac hypertrophy and heart failure is the transverse aortic constriction (TAC) model in the mouse (Rockman et al., 1991; Merino et al., 2018). In this model, pressure overload is produced by aortic ligation and provides a reproducible model of cardiac hypertrophy and gradual heart failure. Several studies have used this model to evaluate protein synthesis pathways and investigate the therapeutic benefit of their modulation.

The mTOR pathway plays an essential regulatory role in cardiovascular physiology and pathology. Both mTORC1 and mTORC2 signaling are crucial for embryonic cardiovascular development and preserving function in the adult (Sciarretta et al., 2018). Specific cardiac ablation of *mTOR* is embryonic lethal and disruption of mTORC1 components postnatally is associated with increased cardiac dysfunction, apoptosis, metabolic changes and heart failure (Sciarretta et al., 2014). It is widely accepted that mTORC1 activation and signaling is required for the development of adaptive hypertrophy and maintenance of heart function in response to pressure overload (Sciarretta et al., 2018). In the absence of mTOR signaling, inadequate remodeling of the heart under increased mechanical strain leads to dilated cardiomyopathy (Zhang et al., 2010). Rapamycin, a potent mTORC1 inhibitor, alleviates established hypertrophy and improves cardiac function following TAC-induced pressure overload in murine models (Shioi et al., 2003; McMullen et al., 2004; Gao et al., 2006). Cardiac hypertrophy promoted by increasing systolic blood pressure in the spontaneously hypertensive rat model could also be attenuated with application of rapamycin (Soesanto et al., 2009). It is important to note, however, that while mTORC1/2 signaling is necessary for cardiomyocyte survival and adaptive hypertrophy in response to mechanical or ischemic trauma, persistent activation of mTOR in a disease setting contributes to pathological hypertrophic remodeling, accumulation of misfolded proteins, energy stress and impaired ventricular and overall heart function (Buss et al., 2009). It has been demonstrated that partial mTORC1 inhibitors are effective in reducing an exaggerated hypertrophic response under pressure overload or chronic myocardial infarction and thereby alleviate tissue damage and heart failure (Shioi et al., 2003). On its own, mTOR signaling is not enough to induce hypertrophy but it is a major contributor and hence has become an attractive target for therapeutic intervention under settings of sustained mechanical stress on the heart (Shen et al., 2008). Partial inhibition of mTORC1 during cardiac stress has been under intense investigation in order to achieve dampening of the maladaptive effects of sustained mTORC1 signaling without disrupting its normal physiological actions. Other studies have investigated the role of components of the mTORC1 complex in the heart under physiological and pathological conditions, such as the adaptor protein Raptor. Mice deficient in myocardial *raptor* display cardiac dysfunction leading to heart failure in response to pressure overload induced by TAC; this is associated with a lack of adaptive cardiomyocyte growth due to reduced protein synthesis (Shende et al., 2011). It has also been demonstrated that cardiac specific overexpression of the gene encoding the mTORC1 endogenous inhibitor, PRAS40, is associated with blunted pathological remodeling after pressure overload and preservation of cardiac function (Völkers et al., 2013a, b).

The role of mTORC2 in cardiac pathology has received considerably less attention. Bénard et al. showed that stromal interaction molecule 1 (STIM1) is required for the initiation of compensatory hypertrophy in response to TAC-induced overload. STIM1 directly activates mTORC2/Akt signaling in order to preserve cardiac function (Bénard et al., 2016). The adaptor protein rictor is unique to and critical for mTORC2 signaling. Cardiomyocyte specific inducible deletion of *RICTOR* leads to cardiac dysfunction in response to pressure overload, again reinforcing the importance of mTOR signaling in the short term, adaptive response to increased mechanical strain (Shende et al., 2016). A similar observation of cardiac dysfunction was seen by Völkers et al. in their *RICTOR* knockdown model which was tested under chronic infarction induced by permanent ligation. In addition to mTOR, the Hippo pathway is another major regulator of cell growth, division and apoptosis. While mTOR signaling promotes growth, the Hippo pathway exerts the opposite effect through negative regulation of its downstream effectors; transcriptional co-activators, yes-associated protein (YAP) and transcriptional coactivator with a PDZ-binding domain (TAZ) (Hansen et al., 2015). The Hippo kinases, MST1/2 in mammals, when active phosphorylate and activate the kinases LATS1/2 which in turn phosphorylate and inactivate YAP and TAZ. When inactive, YAP/TAZ are retained in the cytoplasm where they undergo degradation. When not repressed and in their active form, YAP/TAZ translocate to the nucleus where they predominantly interact with transcription factors from the TEA domain members (TEADs) to promote activation of genes linked with growth (Meng et al., 2016). There has been some research highlighting the cross-talk between Hippo and mTOR signaling during disease states of increased cell growth and proliferation such as cancer (Artinian et al., 2015), however, very little is known with regards to cardiac hypertrophy. One key study has demonstrated that mTORC2 signaling preserves cardiac function following pressure overload induced by TAC by inhibiting the Hippo kinase, MST1 (Sciarretta et al., 2015).

GSK-3β is a negative regulator of protein synthesis and plays a critical role in the cardiomyocyte hypertrophic response to increased mechanical strain. The mechanical stimulus of aortic banding results in a significant decrease in GSK-3β activity which allows for the classic cardiomyocyte hypertrophic response – increased protein accumulation as a result of enhanced protein synthesis, enhanced sarcomere organization and re-expression of the fetal gene program (Haq et al., 2000). Constitutive activation or increased expression of the active form of GSK-3β attenuates pressure overload-induced cardiac hypertrophy *in vivo*, in part due to inactivation of NFAT target genes (Haq et al., 2000; Antos et al., 2001). Active GSK-3β represses eIF2α-mediated protein translation (Antos et al., 2001) and GSK-3β is the primary kinase that phosphorylates eIF2Bε at Serine 535 in rat cardiomyocytes thereby impeding the initiation of translation and resulting in decreased cardiomyocyte hypertrophy (Hardt et al., 2004).

Protein translation rates in the adult heart are generally one of the lowest in the body because cardiomyocytes terminally differentiate soon after birth and therefore show little growth potential and have low cell turnover (Garlick et al., 1980; Paradis et al., 2014). It is only once the heart is mechanically stimulated in an intense and/or prolonged manner e.g., endurance exercise or heart failure, that protein synthesis rates increase, and cardiomyocytes become hypertrophic. One possible mechanism by which biomechanical forces can alter translational control in the heart is via a poly(A) tail based modulatory mechanism. All mature mRNA transcripts in mammalian cells possess a long tail sequence at one end composed of adenosine nucleotide repeats referred to as the poly(A) tail (Hocine et al., 2010). Certain factors can bind onto the poly(A)tail and influence the fate of the mRNA i.e., how efficiently it is translated or degraded (Burgess and Gray, 2010). PABPC1 is a poly(A)tail binding protein known to facilitate mRNA translation (Kini et al., 2016). A recent study by Chorghade, Seimetz and colleagues investigated how PABPC1 mediates protein translation using mouse and human cells. They highlight that PABPC1 is highly expressed in the heart before birth but is downregulated to almost undetectable levels in the adult heart. They found that this decrease in PABPC1 expression was not a result of lower transcription levels but due to changes in translation of the mRNA transcript. The mRNA for PABPC1 has a much shorter poly(A) tail in the adult heart and this affects its translational efficiency causing low protein expression in the adult vs. neonatal heart. This study highlighted that the length of the PABPC1 mRNA poly(A) tail can be extended, and therefore, protein production can be re-established in the adult heart when it has been subjected to hypertrophic conditions triggered by endurance exercise or cardiovascular disease. Experimental re-introduction of PABPC1 in adult hearts allowed for an interaction with pre-initiation factor, eIF4G, which promotes the recruitment of ribosomes and the activation of protein translation (Chorghade et al., 2017).

Impaired force sensing or changes to mechanical signaling that regulates protein translation are clearly linked to cardiac remodeling. Further investigation is required into the mechanical regulation of components of the translational machinery and factors that govern the initiation of protein translation in the context of increased mechanical strain, in both physiological and pathological contexts.

## ER Stress and Biomechanical Stress

The endoplasmic reticulum (ER) plays a crucial role in protein synthesis, folding and quality control to maintain cellular and tissue function (Walter and Ron, 2011). Under pathological mechanical stress such as pressure overload, the tight balance of protein expression and quality control is disrupted, leading to changes in post-translational modifications, increased protein aggregates and misfolding, decreased protein stability and ultimately an ER stress response (Doroudgar et al., 2015). The ER stress response can activate the unfolded protein response (UPR), thus promoting an acute decrease in protein synthesis, increased protein degradation of defective or misfolded proteins and increased synthesis of protective proteins (Glembotski, 2007). These acute mechanisms are cardioprotective in response to dynamic and physiological changes in pressure stimuli, however, their prolonged activation is associated with cardiac cell death (Sun et al., 2015).

**FIGURE 2:**
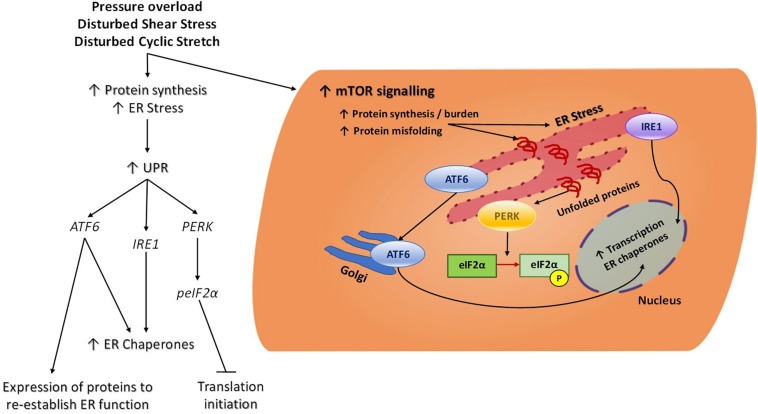
Cellular ER Stress signaling in response to mechanical force. Increased protein synthesis results from chronic mechanical activation of pathways such as mTOR. This results in an increase in protein burden, accumulation of misfolded proteins and ER stress. In an attempt to re-establish ER homeostasis, the UPR is triggered and this consists of three main branches. The eIF2α kinase PERK is an ER transmembrane protein which acts as a sensor to increased protein load and accumulation of unfolded proteins in the ER. Once stimulated, PERK will phosphorylate eIF2α and thereby block global translation initiation to help reduce protein burden in the ER. Phosphorylation of eIF2α also leads to translation of a specific subset of mRNA which will help maintain cellular function and promote cell survival during stress conditions. In times of ER stress, ATF6 will translocate from the ER to the Golgi where it will become cleaved and function as an active transcription factor, promoting the transcription of ER chaperones. IRE1catalyses the splicing of key mRNAs that will become functional transcriptions factors and similar to ATF6, these will promote the transcription and ultimate translation of ER chaperones which will facilitate proper protein folding and degradation to alleviate ER burden. Endoplasmic reticulum (ER), Mammalian target of rapamycin (mTOR), Unfolded protein response (UPR), Eukaryotic translation initiation factor 2-alpha kinase 3, also known as protein kinase R (PKR)-like endoplasmic reticulum kinase (PERK), Activating transcription factor 6 (ATF6), Serine/threonine-protein kinase/endoribonuclease inositol-requiring enzyme 1 (IRE1), Eukaryotic initiation factor 2α (eIF2α).

In response to biomechanical stress, phosphorylation of eIF2α blocks initiation of translation and, as such, helps relieve the excess burden of increased protein synthesis and accumulated unfolded proteins in the ER (see Figure 2) (Doroudgar et al., 2015). Attenuation of eIF2α phosphorylation by cardiomyocyte-specific gene deletion of one of its kinases (PERK) resulted in disruption of the cardiac stress response and exacerbated cardiomyocyte hypertrophy, fibrosis and apoptosis (Liu et al., 2014). While deletion of the gene encoding PERK (*EIF2AK3*) appears to be detrimental in the overloaded heart, gene deletion of other eIFα kinases such as GCN2 (*EIF2AK4*) and PKR (*EIF2AK2*) appears to confer some benefit in pressure overload mouse models. When compared to wildtype mice, GCN2 gene deletion did not reduce the degree of cardiac hypertrophy but did protect against ventricular dysfunction, cardiac fibrosis and apoptosis following pressure overload (Lu et al., 2014). Similarly, PKR gene knockout mice are less prone to pressure overload-induced cardiac fibrosis and have preserved left ventricular function despite displaying similar cardiac hypertrophy to their wildtype littermates (Wang et al., 2014). The molecular mechanisms responsible for the different phenotypes of eIF2α kinase gene knockout mouse models following pressure overload remain incompletely understood, but there is increased appreciation for roles of GCN2, PERK and PKR independent of eIF2α phosphorylation. For instance, PERK is a transmembrane protein spanning the ER membrane and not only reduces protein burden via eIF2α-mediated translation block but has been linked to sensing protein folding interactions in the ER during the unfolded protein response (Donnelly et al., 2013). Therefore, cardiac deletion of the gene encoding PERK would ablate ER homeostasis which the other cytoplasmic eIF2α kinases may not be able to fully compensate for and hence cause a more severe reaction to pressure overload.

Reperfusion of the ischemic heart is essential in order to salvage the myocardium, however, it also imposes mechanical stress and injury to the heart. Ischemia-reperfusion (IR) injury induces marked oxidative stress and intracellular calcium overload, leading to ER stress and activation of the UPR. An important regulator of the UPR in the cardiomyocyte in response to hypertrophy is Activating Transcription Factor 6 (ATF6) (Glembotski, 2014). Upon ER stress, ATF6 is activated and triggers expression of key proteins which will re-establish normal ER function and folding capacity (Figure 2; Martindale et al., 2006). Acute activation of ATF6 protects the heart following I/R injury by reducing necrosis and apoptosis (Jin et al., 2017), however, sustained activation of ATF6 and its pro-apoptotic target genes could have detrimental effects on the heart under pathological mechanical stress (Choi et al., 2016). A possible mechanism by which ATF6 regulates cardiac function in response to biomechanical stress is via Rheb-dependent activation of the mTORC1 signaling pathway and downstream protein synthesis (Blackwood et al., 2019). Rheb regulates mTORC1’s kinase functions and activity such that when Rheb is in its active GTP-bound state it will directly interact with and activate mTORC1. Another mechanism is via regulation of ubiquitination in the stressed myocardium during compensatory and pathological hypertrophy via the ATF6 target gene *Hrd1* (Sun et al., 2015). Targeted suppression of *Hrd1 in vivo* was associated with pronounced pathological hypertrophic remodeling in response to pressure overload, whereas overexpression of *Hrd1* in the heart led to a significant repression in hypertrophy and preserved heart function under pressure overload.

Heat shock proteins and chaperones protect the heart against pathogenic misfolded and accumulated proteins occurring under biomechanical stress (Ranek et al., 2017). Heat shock protein 70 and its protein homolog, heat shock cognate 70 (HSP70 and HSC70, respectively) defend against cardiomyocyte damage by facilitating folding and transport of new proteins and protein degradation at the proteasome. In response to biomechanical stress, HSP70 expression increases in order to alleviate the increased misfolded protein burden. Animal models of inducible HSP70 expression have indicated a cardioprotective role for HSP70 in response to acute cardiac mechanical stress (Bernardo et al., 2016). Interestingly, increased HSP70 expression may only provide protection under acute mechanical stress, such as exercise or I/R, as studies using mice exposed to chronic pressure overload- induced hypertrophy demonstrated no benefit having increased expression of HSP70 (Weeks et al., 2012; Sapra et al., 2014).

Carboxyl terminus of HSC70-interacting protein (CHIP) is expressed in cardiac muscle and functions as a co-chaperone, facilitating the refolding of misfolded proteins either by itself or by mediating its co-chaperones (heat shock proteins HSP70, HS70 and HSP90) (Kettem et al., 2010). CHIP also plays a critical role in protein degradation through its ubiquitin ligase activity therefore it has an essential role in myocardial protein quality control and expression (McClellan and Frydman, 2001). While overexpression or loss of the gene encoding CHIP (*ATCHIP*) does not affect steady state heart function, manipulation of CHIP gene expression levels has profound effects on myocardial function following an increased mechanical load emphasizing the importance of cardiac CHIP levels in preserving heart function under stress (Zhang et al., 2005). CHIP gene KO mouse models exhibit adverse cardiac hypertrophy in response to either exercise or pressure overload as measured by increased cardiomyocyte size, heart weights and wall thickness (Schisler et al., 2013; Willis et al., 2013). Mice with suppressed CHIP expression and subjected to pressure overload had increased mortality rate associated with severe cardiac hypertrophy and fibrosis (Schisler et al., 2013), impaired HSP70 expression (Zhang et al., 2005) and increased mTOR signaling (Dickey et al., 2008), while mechanical stress from MI or I/R injury in CHIP KO mice causes considerably larger, more damaging infarcts and decreased survival.

## Mechanical Forces and Protein Translation in Vascular Cells

### Vascular Smooth Muscle Cells (VSMCs)

VSMCs are the major contractile component of blood vessel walls and experience cyclic strain but are generally shielded from shear stress under physiological conditions (Wang et al., 2018). Endothelial cells (ECs) respond to their mechanical environment and crosstalk with VSMCs in order to maintain vascular tone and mediate vascular remodeling. Under stressed or pathological states where there is vessel injury, the endothelial layer is compromised or endothelial signaling is dysfunctional, such as in hypertension or atherosclerosis. Under such conditions, VSMCs are vulnerable to exposure of shear stress from blood flow or their signaling and function can change as a result of inappropriate EC activation (Scott et al., 2012; Kim et al., 2017). Pathological mechanical trauma and changes to cyclic stretch triggers VSMCs to undergo gene, protein expression and phenotypic changes. Examples of this are decreases in contractile genes such as *SM22*α and those encoding myosin light chain, and increases in cell hypertrophy, proliferation and migration (Huang et al., 1999; Feil et al., 2004; Chiu et al., 2013; Wang et al., 2018). The dysregulated proliferative VSMC phenotype is associated with cardiovascular states where the mechanical environment is perturbed such as pulmonary hypertension and atherosclerosis (Morrell et al., 2009; Bennett et al., 2016).

The mTOR signaling pathway has been shown to be activated in VSMCs in response to cyclic strain (Li et al., 2003) and has since been investigated both *in vitro* and *in vivo* in pathological hypertensive settings. Houssaini et al. induced pulmonary hypertension in rats and observed both mTORC1 and mTORC2 activation which contributed to increased pulmonary artery SMC (PASMC) growth compared to control rats. When they treated the hypertensive rats with rapamycin to inhibit mTORC1 signaling, they observed a decrease in SMC proliferation and reduced vessel remodeling (Houssaini et al., 2013). A more recent study by Tang and colleagues evaluated the contribution of mTORC1 and mTORC2 in the development and progression of pulmonary hypertension in mouse models. They functionally disrupted mTORC1 and mTORC2 specifically in SMCs by knocking out genes encoding the adaptor proteins raptor or rictor, respectively (Figure 1). When they disrupted mTORC1 signaling, in agreement with previous studies, they observed amelioration of SMC proliferation and therefore reduced development of hypertension. In contrast, when they knocked out *RICTOR* and therefore interfered with mTORC2 signaling, this caused spontaneous pulmonary hypertension as a result of upregulation of platelet derived growth factor receptors (Tang et al., 2018). This therefore suggests that mTORC2 confers some protective benefit to the SMC phenotype and vascular remodeling, however, the mechanisms and signaling involved require further clarification especially as other studies have shown mTORC2 plays a key role in proliferation and survival of pulmonary artery SMCs in pulmonary arterial hypertension (Goncharov et al., 2014).

Mechanical forces can stimulate ER stress signaling in VSMCs and chronic activation of this response mediates vascular disease progression such as in atherosclerosis, hypertension and aneurysms (reviewed in Shanahan and Furmanik, 2017). Cheng et al. subjected rat aortic SMCs to cyclic stretch to mimic the hemodynamic environment found in arterial vessels. They found that the downstream target of ER stress transmembrane protein PERK, C/EBP homologous protein (CHOP), was upregulated by cyclic stretch suggesting ER stress activation (Figure 2; Cheng et al., 2008). Another study by Wan et al. suggested that under mechanical stress induced by hypertension, a positive feedback loop is triggered in aortic SMCs whereby increased mechanical trauma activates the ER stress response and this further exacerbates hypertension. The mechanism by which this occurs is increased splicing of the conductance Ca^2+^ voltage activated K^+^ channels which are essential for maintaining vascular tone and contractility (Wan et al., 2015). A complementary study by Liang et al. demonstrated that aberrant ER stress in VSMCs increases their contractility and as such promotes elevated blood pressure; activation of AMPK counteracted high blood pressure by reducing the effects of ER stress *in vivo* and is therefore essential for vascular homeostasis (Liang et al., 2013).

It has been established for some time that ribosomal proteins have extra-ribosomal functions beyond that of the classical biochemistry of protein translation (Wool, 1996; Graifer et al., 2014; Zhou et al., 2015). Ribosomal protein L17 (RpL17) is a component of the large 60S ribosomal subunit but has also been shown to act as a VSMC growth inhibitor. Smolock et al. were first to show that RpL17 expression is inversely correlated with VSMC growth and that *RPL17* depletion promotes VSMC proliferation using a mouse model of partial carotid ligation. This study suggested that RpL17 could therefore represent a potential therapeutic candidate for limiting VSMC proliferation during carotid intima-media thickening (Smolock et al., 2012). It remains to be further investigated how the ribosome-free ribosomal proteins are balanced or coordinated with their traditional roles in protein synthesis and ribosome biogenesis during normal cell growth and proliferation.

### Endothelial Cells (ECs)

There is little study on how mechanical forces from blood flow influence EC function with regards to protein synthesis mechanisms and components of the translational machinery (ribosomes, polysomes, elongation and initiation factors, aminoacyl-tRNA synthetases) and how they could mediate general EC-shear stress responses aside from ER stress in disturbed flow settings. ECs reside in a highly dynamic mechanical microenvironment, and as such, need to be able to adapt quickly changing mechanical stimuli. Translation can occur independently of transcription, suggesting a rationale for specific force-dependent mechanisms that can regulate translation of proteins to bring about rapid cellular responses to force (Brant-Zawadzki et al., 2007). ECs are at the frontline in responding to mechanical cues which alter their activity and phenotype and influence the biologic behavior of the vessel wall i.e., contraction-dilation of blood vessels to mediate changes in blood pressure and re-direct blood flow under exercise training or times of stress (Givens and Tzima, 2016). ECs can also respond to various agonists in the circulation but mechanotransduction, the sensing of a biophysical signal which is converted into an intracellular biochemical response, is more rapid than ligand-receptor signaling (Na et al., 2008). Mechanotransduction responses in ECs involves the dynamic modification of proteins via phosphorylation/de-phosphorylation which will ultimately influence transcriptional and translational control mechanisms. While transcriptional control mechanisms require a longer timeframe to employ, separate translation-only control mechanisms allow ECs to mount a more immediate response to a change in mechanical stimuli, ensuring cell homeostasis while longer term transcriptional changes to gene expression can be put in place.

There is limited study on how fluid shear stress influences protein translation in ECs independent of changes at the transcriptome level. Kraiss et al. were first to demonstrate that fluid shear stress, in the absence of growth factors or hormones, independently activates the mTOR pathway in ECs through phosphorylation of mTOR downstream target, p70S6K (Figure 1). In this same study, the investigators highlighted that FSS can modulate protein expression without changing mRNA levels, again revisiting this idea of the disconnect between mRNA and protein levels as a result of translational control. The activation of p70S6K controls translation of a specific set of mRNA transcripts into protein. One of these is the protooncogene, Bcl-3, which was used in this study to detect changes in protein expression following p70S6K activation by shear stress. They found that Bcl-3 expression was rapidly induced following short-term shear stress and its upregulation was attenuated in the presence of Rapamycin but not in response to actinomycin D, suggesting that upregulation is due to translation and not transcription (Kraiss et al., 2000).

An additional study by Kraiss et al. demonstrated that fluid shear stress can modulate the protein expression of adhesion molecule, E-selectin, on the EC cell surface independent of changes to E-selectin mRNA levels. To further investigate this post-transcriptional mechanism, they recovered the polysome fractions of ECs which had been stimulated to express E-selectin and compared them with pre-stimulated ECs which had then been subjected to shear stress. Fluid shear stress markedly reduced the amount of E-selectin mRNA bound to active polysomes compared to the stimulated only ECs, which had a high level of mRNA associated with actively translating polysomes. To ensure this result was not attributed to a general overall decrease in protein translation following shear stress, they used a radiolabeled methionine incorporation assay to measure relative global protein synthesis occurring in the flowed vs. non-flowed samples. The predominant response of ECs exposed to fluid shear stress shifted toward an overall increase in global protein synthesis compared to their static counterparts therefore indicating a specific downregulation of E-selectin expression. This suggests that the mechanical stimulus of shear stress can influence translational control and specifically control a subset of mRNAs. Interestingly, unlike with Bcl-3, the application of Rapamycin did not affect the flow-mediated response of E-selectin expression suggesting its translation is mTOR/p70S6K independent (Kraiss et al., 2003).

Other studies have examined the effects of different types of shear stress on protein translation mechanisms. Both laminar and disturbed fluid shear stress induce rapid phosphorylation of mTOR at its Serine 2448 and its downstream target p70S6K which is important for protein translation and cell growth. Activation of p70S6K persists under oscillatory shear stress but shows a transient activation following sustained exposure to laminar shear stress (Guo et al., 2007). This highlights the differential actions of different mechanical forces on protein translational signaling. Additionally, mTOR can be also be activated in response to low shear stress as shown by increased phosphorylation of downstream target 4EBP1 (Vion et al., 2017).

In addition to shear stress, pressure also regulates protein translation signaling. Rice et al. investigated pressure-induced activation of p70S6K and other protein synthesis regulators, Akt and GSK3β, in rat aortae from young and aged rats. P70S6K mediates the translation of mRNA transcripts related to cell cycle progression and the translational machinery. They found that pressure-induced phosphorylation of p70S6K and Akt-dependent GSK3β (Serine 9) was attenuated in the aged rat aorta compared to the young adult group suggesting that physiological aging elicits changes to protein synthesis and cell growth pathways (Rice et al., 2005). This study also showed that aged vessels are less compliant as aortic wall thickness increased in the aged animals. More studies are required to highlight the differential mechanisms that occur with normal aging to mechanotransduction pathways in the vasculature which may have different signaling pathways to that which occurs in vascular disease or with age.

The ER stress response, also referred to as the unfolded protein response (UPR), promotes an acute decrease in protein synthesis to ensure ER capacity can match demands of protein load (Walter and Ron, 2011). Activation of the ER stress response also leads to increased protein degradation of defective or misfolded proteins whilst augmenting synthesis of protective proteins (Glembotski, 2007). Protein misfolding is a result of increased protein synthesis, changes in protein oxidation, post-translational modifications and decreased proteasome capacity (Sun et al., 2015). In regions of disturbed flow, such as the inner curvature of the aortic arch, ER stress activates adaptive UPR signaling (see Figure 2; Davies et al., 2013). A study by Civelek et al., 2009 observed upregulated gene expression of *ERN1* (IRE-1) and *ATF6*, both transducers of the UPR response, in the aortic arch compared to the descending aorta. This stress response in the aortic arch could be persistently active in order to mitigate the effects of the imbalanced mechanical environment which promotes sustained protein translation and contributes to the accumulation of pathological levels of misfolded proteins. Further analysis of differential effects of shear stress on the EC ER stress response was performed by Bailey and colleagues. They subjected human aortic ECs to low and high shear stress and examined the levels of key factors involved in the ER stress response. They found that low shear stress (2 dynes/cm^2^) induced high expression of eIF2α and Xbp1 and high shear stress (12 dynes/cm^2^) was associated with low Xbp1 expression (Bailey et al., 2017). This data coincides with Zeng et al. who found that Xbp1 was highly expressed in areas of the vasculature which are susceptible to disturbed flow patterns e.g., branch points. In addition, there were similar high and low expression patterns in their *in vitro* studies when analyzing disturbed and laminar flow, respectively (Zeng et al., 2009).

### Perspectives

The control of protein expression in the cardiovascular system is incredibly sensitive to the effects of mechanical forces. Protein translation in the heart is relatively low unless mechanical signaling increases substantially and/or is sustained which can be of a physiological or pathological nature. Endurance exercise causes increases in hemodynamic forces which, if sustained, will trigger protein synthesis mechanisms and adaptive cardiac hypertrophy to cope with increased mechanical demands. In conditions where hemodynamic forces are perturbed, such as hypertension, atherosclerosis and heart failure, dysregulated protein synthesis can contribute to worse outcomes in heart and vessel function and disease progression. These can include dysregulation of mTOR signaling or components which modulate protein translation initiation, such as eIF2α. Signaling systems such as those involved in the ER stress response are highly mechanosensitive and help to regulate protein burden in conditions of mechanical disturbance. Pharmacologic and/or genetic inhibition of protein translation pathways has been shown to extend life span in mammals and reduce cardiac remodeling and heart failure in response to increased biomechanical stress. These studies suggest that targeting of protein translation pathways, especially when they are aberrantly activated in conditions of mechanical disturbance, may represent a novel therapeutic strategy to confer cardioprotection and vessel homeostasis.

## Author Contributions

LS, JR, and ET wrote the review.

## Conflict of Interest

The authors declare that the research was conducted in the absence of any commercial or financial relationships that could be construed as a potential conflict of interest.
